# Haplotypes at the *Tas2r *locus on distal chromosome 6 vary with quinine taste sensitivity in inbred mice

**DOI:** 10.1186/1471-2156-6-32

**Published:** 2005-06-06

**Authors:** Theodore M Nelson, Steven D Munger, John D Boughter

**Affiliations:** 1 Anatomy and Neurobiology, University of Maryland School of Medicine, Baltimore, Maryland, 21201, USA; 2 Program in Neuroscience, University of Maryland School of Medicine, Baltimore, Maryland, 21201, USA; 3 Anatomy and Neurobiology, University of Tennessee Health Science Center, Memphis, TN, 38163, USA

## Abstract

**Background:**

The detection of bitter-tasting compounds by the gustatory system is thought to alert animals to the presence of potentially toxic food. Some, if not all, bitter stimuli activate specific taste receptors, the T2Rs, which are expressed in subsets of taste receptor cells on the tongue and palate. However, there is evidence for both receptor-dependent and -independent transduction mechanisms for a number of bitter stimuli, including quinine hydrochloride (QHCl) and denatonium benzoate (DB).

**Results:**

We used brief-access behavioral taste testing of BXD/Ty recombinant inbred (RI) mouse strains to map the major quantitative trait locus (QTL) for taste sensitivity to QHCl. This QTL is restricted to a ~5 Mb interval on chromosome 6 that includes 24 genes encoding T2Rs (*Tas2rs*). *Tas2rs *at this locus display in total 307 coding region single nucleotide polymorphisms (SNPs) between the two BXD/Ty RI parental strains, C57BL/6J (quinine-sensitive) and DBA/2J (quinine insensitive); approximately 50% of these mutations are silent. Individual RI lines contain exclusively either C57BL/6J or DBA/2J *Tas2r *alleles at this locus, and RI lines containing C57BL/6J *Tas2r *alleles are more sensitive to QHCl than are lines containing DBA/2J alleles. Thus, the entire *Tas2r *cluster comprises a large haplotype that correlates with quinine taster status.

**Conclusion:**

These studies, the first using a taste-salient assay to map the major QTL for quinine taste, indicate that a T2R-dependent transduction cascade is responsible for the majority of strain variance in quinine taste sensitivity. Furthermore, the large number of polymorphisms within coding exons of the *Tas2r *cluster, coupled with evidence that inbred strains exhibit largely similar bitter taste phenotypes, suggest that T2R receptors are quite tolerant to variation.

## Background

Animals use the gustatory system to provide information about food quality. For example, sweet-tasting foods may have a high caloric content and are preferred, while bitter-tasting foods often contain toxic substances, and are generally avoided. Two families of G protein-coupled receptors (GPCRs) expressed in subpopulations of taste receptor cells (TRCs) of the gustatory epithelium have been implicated in the detection and transduction of sweet, bitter and umami (i.e., glutamate) taste: T1Rs for sweet-and umami-tasting stimuli [1-8], and T2Rs for bitter-tasting compounds [9-11].

The genes that encode T2Rs, the *Tas2rs*, were first identified by database mining of mammalian genomes near chromosomal markers previously linked to differences in bitter taste sensitivity [9,11-18]. In mice, the majority of *Tas2rs *lie within a single cluster on distal chromosome 6. Thirty-three human *Tas2rs *(including 8 pseudogenes) and thirty-six mouse *Tas2rs *(including 3 pseudogenes in C57BL/6J mice) have been identified [9,11,19], and several of these respond to particular bitter stimuli in heterologous expression assays [10,20-23], or represent a strong candidate gene for a specific bitter taste quantitative trait [18,24,25].

Several quantitative trait loci (QTL) have been identified that influence two-bottle intake of bitter stimuli in the mouse, including loci for quinine (*Qui*) [12,16,26], cyclohexamide (*Cyx*) [13] and sucrose octaacetate (*Soa*) [14,15,17] sensitivity. Each of these QTL map to mouse distal chromosome 6 and are linked to the marker *D6Mit13*, which lies within a cluster of 24 intact *Tas2rs *in the C57BL/6 genome (e.g., [16,27,28]). However, the interpretation of these studies remains problematic for two reasons. First, the density of chromosomal markers and number of recombinant inbred (RI) strains used in these earlier studies did not permit the physical definition of the intervals containing each QTL. Second, these previous attempts to map bitter taste QTLs relied on behavioral assays that measured consumption, and were thus susceptible to contributions of post-ingestive effects such as toxicity. As we have shown previously, such effects can confound the quantification of bitter taste behaviors [29]. Therefore, the relevance and/or contribution of the aforementioned QTLs to bitter taste remain unclear.

Furthermore, a number of physiological studies have suggested that the transduction of some amphiphilic bitter compounds, such as quinine and denatonium benzoate, may stimulate taste receptor cells independently of GPCRs (e.g. [30]). Quinine may directly activate G proteins, and both quinine and denatonium can block K^+ ^channels [31-36] ; caffeine, another bitter-tasting substance, directly inhibits intracellular phosphodiesterase [33]. However, the relative contributions of T2R-dependent and T2R-independent mechanisms to the detection of these bitter stimuli are unknown.

Here we use a taste-salient brief-access lick test [29,37] to measure taste sensitivities in C57BL/6J (B6), DBA/2J (D2) and BXD/Ty (BXD) recombinant inbred (RI) mice to two bitter stimuli, quinine hydrochloride (QHCl) and denatonium benzoate (DB). Using 17 BXD lines that were genotyped at 762 informative chromosomal markers, we mapped a major QTL for QHCl taste to a ~5 Mb interval on distal chromosome 6 that contains all 24 of the *Tas2r *genes in the distal cluster. We analyzed the sequence of each *Tas2r *allele in the parental strains (B6 and D2) and 29 RI lines. This analysis revealed that all 24 genes are polymorphic between the two strains, and that these 24 *Tas2rs *comprise a single haplotype that correlates with QHCl taste sensitivity.

## Results

### Taste testing

**Figure 1:**
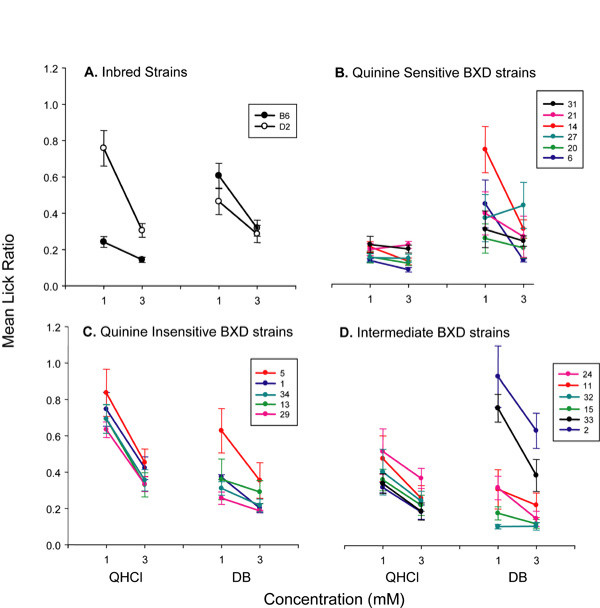
Lick ratios (mean ± SE) for B6, D2 and BXD strains. **(A) **Mean lick ratios for B6 (filled circles) and D2 (open circles) mice at two concentrations of QHCl and DB. In all panels, a lower mean lick ratio indicates a greater aversion, and therefore greater taste sensitivity, to the stimulus. For panels B, C, and D, each BXD strain is represented by a different color, and listed in order from least sensitive to most sensitive to 1 mM QHCL. **(B) **Mean lick ratios for the six BXD strains that are most sensitive to QHCl in this assay. **(C) **Mean lick ratios for the five BXD strains that are least sensitive to QHCl in this assay. **(D) **Mean lick ratios for the six BXD strains intermediate in QHCl taste sensitivity to those in (B) and (C). Cutoffs for the three QHCl taster groups were arbitrarily set, as there was a continuity of the phenotype at 1 mM QHCl: sensitive strains exhibited a lick ratio for 1 mM QHCl of ≤ 0.3, intermediate strains from 0.31-0.6, and insensitive strains > 0.6. The absence of two distinct phenotypic classes suggests that sensitivity to QHCL is under polygenic control.

Previous efforts to map QTL for bitter taste have utilized consumption tests that may be confounded by the contributions of post-ingestive effects [29]. We used a modified brief-access lick test, which minimizes the contribution of such effects [29,37] to determine whether B6 and D2 mice display differences in taste sensitivity to the taste stimuli QHCl and DB. After initially screening B6 and D2 mice to determine stimulus concentrations that were aversive but not saturating [47], we selected two ligand concentrations for each compound that best differentiated the two strains. Subsequent taste testing of BXD RI lines was restricted to these two concentrations (1 and 3 mM for both QHCl and DB). Avoidance by male and female B6 and D2 mice increased (as indicated by the decreased lick ratio) in a concentration-dependent manner for both compounds (Figure 1A; Table 1). There was a significant strain difference for both 1 and 3 mM QHCl (F[1,25] > 24.6; p < 0.0001). D2 mice displayed decreased aversion relative to B6 mice at both concentrations. On the other hand, the strains did not significantly differ in taste sensitivity to DB (Figure 1A). There were no significant effects of gender.

**Table 1:** Mean lick ratios for B6, D2 and BXD mice.

**Strain**	**n**	**Water licks/5s**	**1 mM DB**	**3 mM DB**	**3 mM PR**	**10 mM PR**	**1 mM QH**	**3 mM QH**
**B6**	16	29.02 ± 1.4	0.606	0.318	0.311	0.164	0.241	0.144
**D2**	12	33.91 ± 2.4	0.465	0.286	0.616	0.429	0.758	0.305
**BXD1**	5	32.48 ± 1.5	0.372	0.202	0.314	0.252	0.746	0.422
**BXD2**	4	36.50 ± 0.9	1.025	0.605	0.208	0.230	0.320	0.215
**BXD5**	5	37.23 ± 2.7	0.628	0.354	0.502	0.636	0.836	0.452
**BXD6**	5	22.43 ± 3.5	0.442	0.132	0.200	0.124	0.130	0.080
**BXD11**	7	34.48 ± 3.9	0.310	0.223	0.387	0.260	0.479	0.260
**BXD13**	5	27.74 ± 3.4	0.360	0.290	0.472	0.168	0.692	0.330
**BXD14**	7	31.09 ± 3.5	0.741	0.304	0.293	0.174	0.206	0.126
**BXD15**	5	37.36 ± 1.5	0.178	0.120	0.468	0.198	0.362	0.222
**BXD20**	5	31.26 ± 2.7	0.252	0.200	0.162	0.128	0.150	0.116
**BXD21**	6	19.77 ± 1.5	0.390	0.262	0.313	0.133	0.192	0.217
**BXD24**	5	33.03 ± 2.4	0.318	0.148	0.490	0.238	0.518	0.370
**BXD27**	5	33.46 ± 2.5	0.364	0.434	0.216	0.126	0.146	0.142
**BXD29**	3	40.19 ± 1.5	0.257	0.187	0.657	0.173	0.633	0.333
**BXD31**	5	19.45 ± 0.8	0.302	0.238	0.152	0.158	0.218	0.194
**BXD32**	6	27.22 ± 2.3	0.105	0.107	0.385	0.203	0.405	0.245
**BXD33**	6	29.03 ± 2.2	0.757	0.387	0.377	0.237	0.342	0.188
**BXD34**	6	23.01 ± 3.4	0.310	0.217	0.843	0.257	0.693	0.355

The number of individual mice tested for each strain (n) is listed in the second column. Subsequent columns show the mean lick rate to water during testing (± SEM), mean lick ratio for denatonium benzoate (DB), PROP (6-n-propylthiouracil; PR) and quinine hydrochloride (QH) at each of two concentrations (see Methods for details).

We next tested mice from 17 BXD lines in the same manner. BXD mice also typically avoided both stimuli in a concentration dependent manner (Figures 1B–1D; Table 1). However, QHCl and DB taste sensitivity vary independently across these RI strains: some strains highly sensitive to QHCl are relatively insensitive to DB, and vice versa (Figures 1B–1D).

### QTL mapping

**Figure 2:**
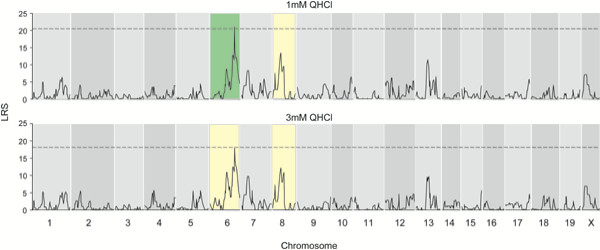
A major QTL for QHCl taste on mouse chromosome 6. **(Top panel) **The interval map (see Methods) shows a significant QTL on chromosome 6 (green) and a suggestive QTL on chromosome 8 (yellow) affecting taste responses to 1 mM QHCl. **(Bottom panel) **For 3 mM QHCl, both QTL were suggestive (yellow). The dashed line indicates genome-wide significance.

Linkage analysis was conducted using Map Manager QTX (version 0.30[38]). No significant QTLs were identified for DB taste sensitivity, although several associations with markers on chromosomes 2,8 and 12 were "suggestive" (LRS > 9.4, genome-wide p = 0.65; see Additional File 1). A significant (LRS > 20.5; genome-wide p = 0.05) QTL for sensitivity to 1 mM QHCl was indicated on chromosome 6, with a second, suggestive (LRS > 11.4; genome-wide p = 0.65) QTL on chromosome 8 (Figure 2A); at 3 mM QHCl, both of these QTL were suggestive (LRS > 10.9) but did not reach genome-wide significance (Figure 2B).

**Figure 3:**
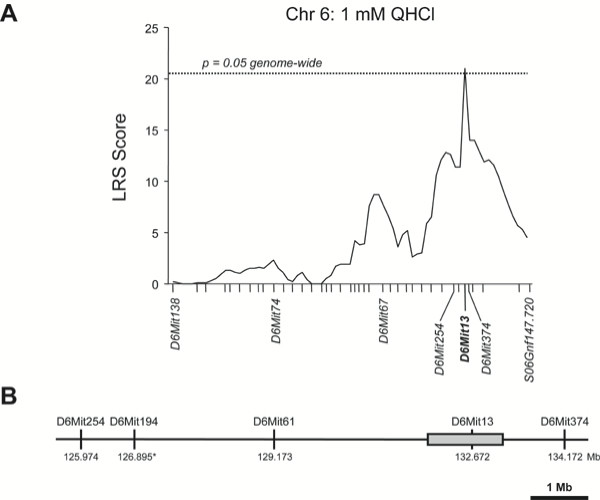
The QHCl QTL is linked to a single marker on chromosome 6. **(A) **As shown in the interval map for chromosome 6, the trait value (lick ratio for 1 mM QHCl) correlates strongly across BXD RI strains with the polymorphic marker D6Mit13 (bold). The dashed line indicates genome-wide significance. (B) The QHCl QTL (which lies between unlinked markers D6Mit61 and D6Mit 374) contains a cluster of putative bitter taste receptor genes, the *Tas2rs *(gray box). Physical positions of the polymorphic markers are given in Mb, and are based on the May, 2004 build of the B6 mouse genome. The physical position of D6Mit194 (*) is tentative.

The chromosome 6 QTL was linked to a single marker, D6Mit13 (Table 2, Figure 3). Adjacent proximal markers D6Mit254 and D6Mit194 are unlinked to the QHCl QTL, as is distal marker D6Mit374. Across the 17 RI lines tested there is at least one recombination event between D6Mit13 and either D6Mit254 (and D6Mit194, the physical position of which is not well defined) or D6Mit374. An additional proximal marker, D6Mit61, which lies between D6Mit194 and D6Mit13, was identified from genotypes of the BXD lines reported by the Jackson Laboratories. BXD/Ty-34 RI mice display a clear D2 phenotype for QHCl taste (Figure 1C) and D2 genotype for D6Mit13, but have a B6 genotype for D6Mit61 [39,40], indicating that D6Mit61 is unlinked to the QHCl QTL. Therefore, this QTL interval can be conservatively defined as that portion of mouse chromosome 6 that lies between D6Mit254 and D6Mit374, but is most likely restricted to the region between D6Mit61 and D6Mit374.

**Figure 4:**
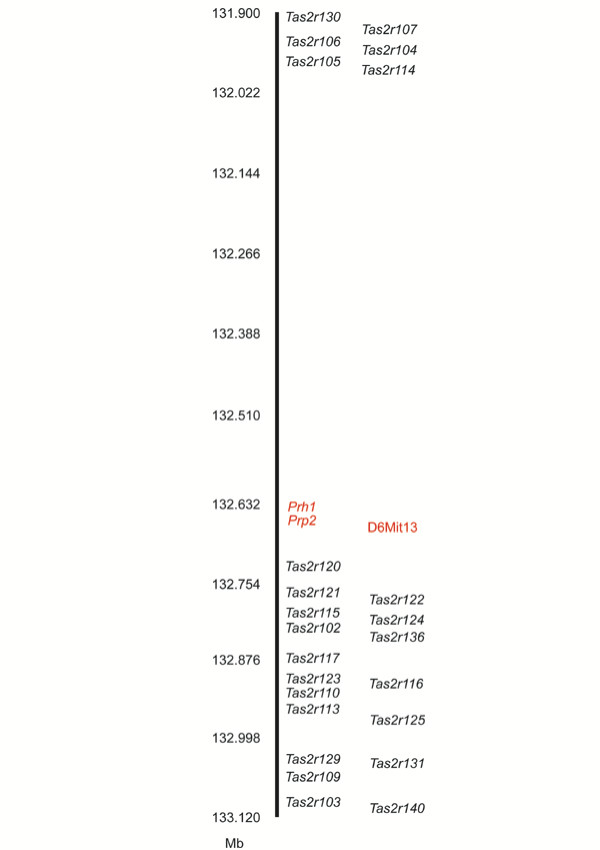
A map of the distal chromosome 6 *Tas2r *cluster. Twenty-four intact *Tas2r *genes map to distal chromosome 6 (black). The *Tas2r*s are found in two subclusters on either side of the polymorphic marker *D6Mit13 *(red) and two genes encoding proline-rich salivary proteins (*Prp2 *and *Prh1*; red). Map positions, in Mb, represent chromosome 6 positions in the May, 2004 assembly of the B6 genome.

Physical mapping of the single linked marker, D6Mit13, and the two closest unlinked markers, D6Mit61and D6Mit374, was performed *in silico *based on the May, 2004 build of the public B6 genome. Based on these marker positions, the size of the QHCl chromosome 6 QTL is less than 5.0 Mb (Figure 3). This region contains a number of known genes, all but eleven of which encode members of two large receptor families: natural killer cell lectin-like receptors, and T2R-type taste receptors. The *Tas2r *genes (which encode the T2Rs) are found clustered within a 1.2 Mb interval on either side of D6Mit13 (Figure 3, Figure 4). Because of their proximity to the linked marker, their demonstrated expression in taste receptor cells, and their role in the detection of at least some bitter-tasting compounds, we hypothesized that one or more of the 24 *Tas2rs *at this locus were responsible for the major QHCl taste sensitivity QTL.

### T2R alleles

If one (or more) *Tas2rs *underlie the chromosome 6 QHCl taste sensitivity QTL, we would predict that one (or more) *Tas2r *genes would exhibit one of three likely characteristics: (1) A *Tas2r *allele is a pseudogene, or is deleted, in D2 (QHCL-insensitive), but not B6 (QHCl-sensitive), mice; (2) Missense mutations in the single coding exon of a D2 *Tas2r *allele impact protein functions such as ligand binding or receptor coupling to downstream signaling cascades; (3) Mutations in noncoding or regulatory regions of a D2 *Tas2r *allele affects expression of the protein product. Though we considered all three of these to be valid possibilities, we initially focused on the likelihood that deletion or mutation within the coding sequence of a single D2 *Tas2r *would correlate with the QHCl taste insensitivity phenotype.

**Figure 5:**
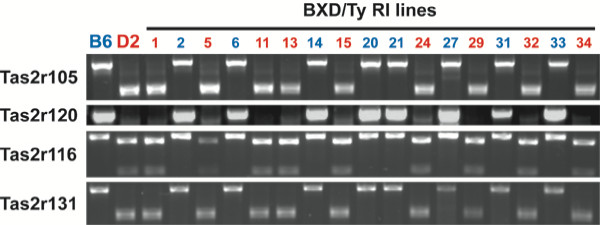
Allelic variation across strains for four *Tas2rs*. B6 and D2 alleles of four *Tas2rs *can be differentiated based on diagnostic restriction digests of amplified PCR products (*Tas2r105*, *Tas2r116 *and *Tas2r131*) or on the presence or absence of a PCR product (*Tas2r120*). In each of the 17 BXD strains tested, *Tas2r* genotype was always correlated with QHCl taster phenotype (blue = B6 taster phenotype, red = D2 taster phenotype). See additional file 1: Table 3 for restriction enzymes and oligonucleotides.

Twenty-four intact *Tas2rs*, along with three apparent *Tas2r *pseudogenes, have been identified in the distal chromosome 6 cluster of B6 mice [19] (Figure 4). We designed oligonucleotides to non-coding regions flanking the coding sequence of each intact *Tas2r *[see Additional file 2]. Using these oligos, we amplified each *Tas2r *coding sequence from D2 genomic DNA. PCR products were subcloned into cloning vectors and sequenced. Comparisons of the sequences of B6 and D2 orthologues revealed that only two of the twenty-four *Tas2r *alleles examined, *Tas2r106 *and *Tas2r124*, were identical across strains at the amino acid level (data not shown). A third, *Tas2r120*, could not be amplified from D2 genomic DNA (Figure 5) using either of two pairs of oligonucleotides (Additional file 2), suggesting that this *Tas2r *is deleted in D2 mice. Two D2 alleles, *Tas2r103 *and *Tas2r117*, contained numerous missense mutations and small deletions that create frame shifts and premature termination; these two genes may be pseudogenes in this strain. The remaining 19 *Tas2r*s contained between one and 16 missense mutations. All 24 *Tas2rs *examined have different alleles in B6 and D2 mice, and 307 single nucleotide polymorphisms are present within coding exons (data not shown). Although polymorphic residues between B6 and D2 *Tas2rs *are found in all regions of the receptors, 23% of the amino acid changes seen are within the first two extracellular loops of the T2Rs (data not shown).

The variability between orthologous receptors in these two inbred strains suggested that it might be possible to narrow the physical boundaries of the QHCl taste QTL by determining which *Tas2r *alleles are correlated with QHCl taste sensitivity. Therefore, we proceeded to screen genomic DNA from 29 available BXD RI lines, including the 17 that we had used in taste testing, for the *Tas2r *alleles they contained. In most cases, we were able to identify diagnostic restriction endonuclease digests that would allow us to quickly identify whether a particular *Tas2r *PCR product was amplified from a B6 or D2 allele. We did not analyze three genes (*Tas2r104*, *Tas2r114 *and *Tas2r110*) where no diagnostic restriction endonuclease could be identified. For *Tas2r120*, which is likely deleted in D2 mice, the absence of a PCR product was diagnostic of the D2 genotype for this gene.

**Figure 6:**
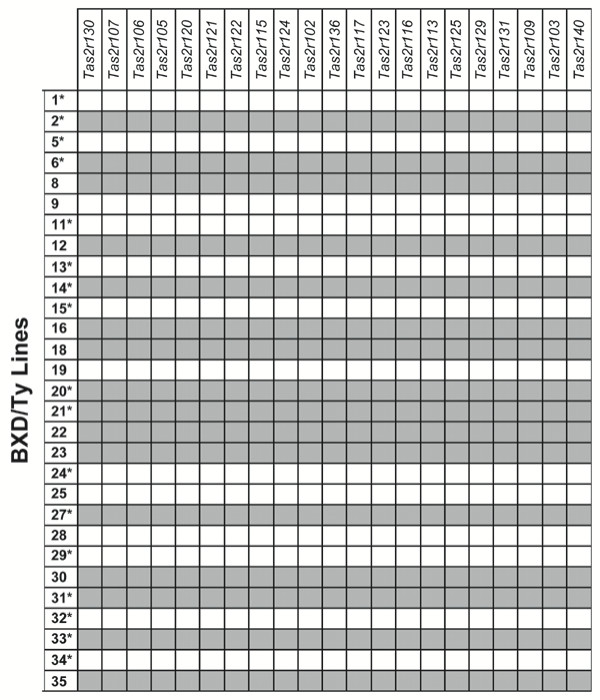
The *Tas2r* cluster is a single haplotype in BXD/Ty RI mice. The coding exon of each of 21 *Tas2rs *in the distal chromosome 6 cluster was amplified genomic DNA from 29 BXD/Ty RI strains. Each *Tas2r *within an individual BXD strain originated from the same parental strain (B6 allele = gray, D2 allele = white). The 17 BXD strains that were behaviorally tested in this study are indicated (*).

**Figure 7:**
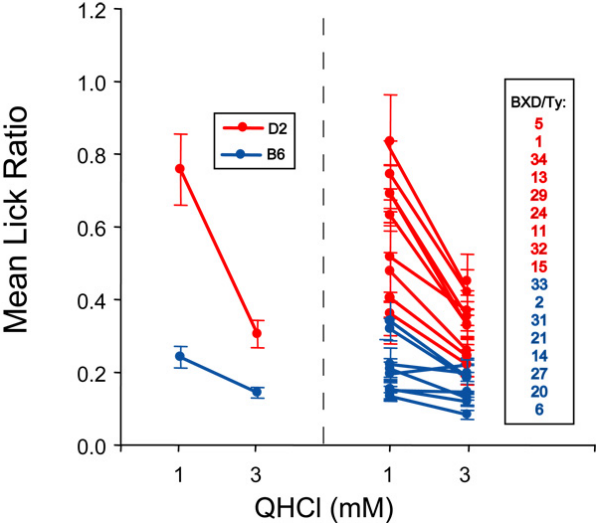
*Tas2r *genotype correlates with QHCl taste phenotype. Mean lick ratios of B6, D2 and BXD strains reported in Figure 1 are grouped based on *Tas2r *haplotype (B6 haplotype = blue, D2 haplotype = red). B6 mice (blue line on left panel) are more sensitive to 1 mM and 3 mM QHCl than are D2 mice (red line on left panel) in brief access taste tests. Similarly, BXD strains with the B6 *Tas2r *haplotype (blue lines, right panel) are more sensitive to QHCl than are BXD strains with the D2 *Tas2r *haplotype (red lines, right panel). The BXD strains are listed in order from least to most sensitive to 1 mM QHCL.

Surprisingly, we discovered that there have been no apparent recombination events within the distal chromosome 6 cluster during the generation of the BXD RI lines. For all RI lines tested, every *Tas2r *within an individual RI line originated from the same parental strain (Figures 5, 6). Furthermore, the genotype of each *Tas2r *gene always correlated with the QHCl taste phenotype (Figures 6, 7), suggesting that the entire *Tas2r *cluster is a single haplotype that varies with QHCl taster status.

## Discussion

The gustatory system of mammals is thought to detect thousands of chemically-diverse bitter-tasting substances [41]. Although specific receptors, enzymes and channels have been implicated in the transduction of bitter stimuli, how interactions of bitter stimuli with taste receptor cells lead to cellular activation and signaling to the central nervous system is still poorly understood. We have found that a single QTL on distal chromosome 6 accounts for most of the variation in QHCL taste sensitivity between B6 and D2 mice. This QTL maps to the same chromosomal position as a previously identified QTL for quinine intake, *Qui *[16,28], indicating that taste is the major factor in regulating quinine aversion. This is an important distinction, as the consumption of bitter-tasting stimuli can be dependent on factors independent of taste, such as toxicity [29].

Using 17 RI lines and 762 chromosomal markers, we have restricted the quinine taste QTL to a < 5 Mb region on distal chromosome 6 that contains 24 *Tas2r *genes. At least 60 other genes also lie within this interval, including two genes that encode proline-rich salivary proteins, *Prp2 *and *Prh1*; these proteins appear to play no direct role in bitter taste [48]. *Tas2rs *are the most likely candidates for the QHCl quantitative trait gene(s) due to: (1) their expression in taste receptor cells and (2) genetic and functional evidence linking them to the detection of a number of bitter taste stimuli. As of yet there is no evidence for quinine activation of T2Rs from functional assays of these receptors in heterologous cells or membrane preparations, likely due to the lipophilic nature of quinine [23]. However, several physiological studies have suggested that quinine can directly activate G proteins or cationic conductances, or can block K^+ ^channels in taste receptor cells [34-36]. While our data indicates that quinine taste is largely T2R-dependent, it is not exclusively so. For example, the BXD RI lines exhibited a range of quinine sensitivity, with several strains having similar sensitivities to that of B6, some strains with sensitivities similar to that of D2, and a third group with a more intermediate phenotype (Figures 1, 7). This observation is consistent with a polygenic basis for quinine taste [16,26]. Also, a suggestive QTL on chromosome 8 does not contain any *Tas2r *genes, but does contain a number of genes encoding ion channels, enzymes and members of other receptor families (our unpublished data). It will be interesting to determine whether this suggestive QTL is linked to quinine taste and, if so, whether it is specific for this single bitter stimulus or more broadly related to all bitter taste.

Of the 29 BXD RI lines examined, there was no apparent recombination event within the chromosome 6 *Tas2r *cluster. While increasing the number of BXD RI lines or the number of markers used for genotyping them would facilitate the definition of smaller QTL intervals, in this case such an effort is unlikely to permit the identification of one or a few *Tas2rs *involved in quinine taste. For example, we examined six lines of AXB and BXA RIs with reported recombinations around D6Mit13; a small sampling of the *Tas2rs *in these RI lines again indicated no recombinations within the *Tas2r *cluster (data not shown). Behavioral genetic approaches have been invaluable for identifying genes involved in taste function, such as the *Tas1r3 *gene that encodes a receptor important for sweet and umami taste [42]. Positional cloning also permitted the identification of the *Tas2r *responsible for the majority of variance of phenylthiocarbamide (PTC) taste sensitivity in humans [18]. In both of these cases, however, the genes linked to saccharin or PTC taste were not tightly clustered with paralogues. For bitter taste, behavioral genetic approaches may be more useful for identifying genes encoding downstream signaling molecules or components of T2R-independent transduction mechanisms. For example, a QTL for PROP avoidance has been suggested on chromosome 7 [16], and we observe a suggestive QTL for quinine taste on chromosome 8 (Figure 2); in neither case are *Tas2rs *found at these loci (data not shown).

It is somewhat puzzling that 22 of the 24 *Tas2rs *examined encode variant proteins in B6 and D2 mice even though these strains exhibit similar taste responses to bitter compounds such as DB or cyclohexamide [47]. Taken together, these observations suggest that *Tas2rs *are quite tolerant of variation, and that perhaps most of the differences observed do not affect domains important for ligand interactions or receptor-mediated signaling mechanisms. Interestingly, 23% of missense mutations in D2 *Tas2rs *affect the first two extracellular loops of the receptors. These two loops have been recently shown to affect the ligand response profiles of some T2Rs [23]. More systematic analyses of structure-function relationships between these T2R variants and an array of bitter stimuli are necessary to determine which changes may impact ligand binding, interactions with other proteins, or overall receptor structure.

Such large numbers of nonsynonymous substitutions between orthologues is suggestive of adaptive selection. Analysis of sequence diversity of *Tas2rs *in humans, great apes and old world monkeys suggest that *Tas2rs *are subject to some degree of positive selection [43,44]. However, the fact that these two mouse strains, members of the same species, are so closely related makes this explanation problematic. It is possible that B6 and D2 mice, which have a similar origin in the early 20^th ^century, inherited different *Tas2r *haplotypes present in wild mouse populations prior to inbreeding. Characterization of *Tas2r *sequences of several wild mouse species or subspecies, or in other inbred lines, would shed light on this issue.

## Conclusion

In conclusion, we have found that sensitivity to the bitter-tasting substance quinine, as assayed by a taste specific brief-access test, is a polygenic trait in mice. However, the major mechanism for quinine taste transduction is likely dependent on one or more T2R receptors. Most *Tas2r *genes in the distal chromosome 6 cluster are polymorphic across inbred strains of mice, and this cluster forms a single haplotype that correlates with quinine taste sensitivity. The numerous differences in T2R protein sequence between these two mouse strains suggests that T2Rs are broadly tuned receptors quite tolerant to sequence variation. This tolerance may help to preserve the ability of T2R-expressing, bitter-sensitive taste cells to respond to a wide array of potentially toxic stimuli.

## Methods

### Mice and solutions

A total of 188 adult male and female mice were behaviorally tested in these experiments: 16 C57BL/6J (B6; 9 males, 7 females), 12 DBA/2J (6 females, 6 males), and 90 BXD/Ty recombinant inbred mice (average = 5 / line; 64 males, 26 females) from 17 unique lines (1, 2, 5, 6, 11, 13, 14, 15, 20, 21, 24, 27, 29, 31, 32, 33, 34). All mice were either obtained from Jackson Laboratories (Bar Harbor, ME), or were bred from mating pairs at UTHSC. At time of testing, mice were individually housed in standard shoebox cages with woodchip bedding and ad libitum food (Teklad 8640 rodent diet). Taste stimuli used in this experiment were made from reagent-grade chemicals: Sucrose, denatonium benzoate, 6-n-propylthiouracil and quinine hydrochloride (Sigma Aldrich Corp.; St. Louis, MO). Concentrations of each solution were made fresh daily using distilled water, and all taste stimuli were presented at room temperature. All animal protocols were approved by the UTHSC Institutional Animal care and Use Committee.

### Brief-access tests

All behavioral tests were conducted in the commercially available Davis MS-160 gustometer (DiLog Instruments, Inc., Tallahassee FL). Testing procedures were similar to those described earlier [29,37]. Briefly, after 24 hours of water deprivation, naïve mice are given a single 20-minute trial consisting of access to a single bottle of distilled water (sipper tube training). On day 2, mice could initiate up to sixteen 5 s trials with a single lick to one of four bottles containing distilled water (trial training). Testing occurred in sessions 3 and 4, with one test session per day per mouse. Trials were 5 s in length with an inter-trial interval of 10 s, and mice had up to 120 s to initiate a trial; if a trial was not initiated during this interval, the shutter closed for 10 s and the next trial was presented. Mice were tested with 2 concentrations each of 4 different taste stimuli [1 and 3 mM QHCl, 1 and 3 mM DB, 3 and 10 mM PROP (unpublished data), and 0.01 and 0.1 M sucrose]. Stimulus trials were presented in 3 blocks of 8 trials, for a total of 24 possible trials per test session. Each block consisted of each concentration of stimulus plus four presentations of distilled water in random order. Individual mice were also tested in random order.

The dependent measure for each computed for each mouse was the *lick ratio *(average number of licks to stimulus_*x *_/average number of licks to water) where *x *is a given concentration of stimulus and the average number of licks to water is derived from the water trials during both test sessions. Lick ratio data for each stimulus were compiled for all individual mice, and means were prepared for each strain. B6 vs. D2 comparisons (Fig. 1A) were made using main effects ANOVA. Lick ratios for individual mice to sucrose were generally ~1.0 (data not shown), indicating that either concentration of this compound was licked at a similar rate to water by these thirsty animals. This stimulus was intended as a "neutral" stimulus, albeit one that has different sensory properties than water, and therefore not analyzed further. This was done to encourage sampling on "non-water" trials, as there is some evidence that mice detect distilled vs. adulterated water in brief-access taste tests based on olfactory clues; there is no evidence that mice can detect or distinguish among concentrations of a particular stimulus [37].

### QTL mapping

Linkage analysis was conducted on BXD mice using freely available software (Map Manager QTX [38]), and BXD genotype data shared by Robert W. Williams, University of Tennessee Health Science Center [45]. Simple interval mapping was conducted. This method evaluates the association between trait values (lick ratios) and expected genotype of a hypothetical quantitative trait locus (QTL) at multiple analysis points between each pair of adjacent marker loci. The significance of each potential association is measured by the likelihood ratio statistic (LRS; e.g. [46]). Permutation analysis (x2000) was used to determine genome-wide significance criteria for LRS scores. Significance was set at p < 0.05 and suggestive refers to p < 0.63. Additional markers used to refine the QTL on chromosome 6 were identified from the Jackson Laboratories online resources for the BXD RI strains [40].

### Identification of T2R alleles

Oligonucleotides were based on published *mTas2r *B6 or 129/SvJ cDNA sequences or on the public B6 genome. Entire coding regions plus ~50 kb of flanking sequence of each single-exon *Tas2r *was amplified from D2 or BXD RI genomic DNA (Jackson Laboratories, Bar Harbor, ME) by polymerase chain reaction (PCR) using a high-fidelity polymerase TaqPro Complete (Denville Scientific, South Plainfield, NJ). PCR products were subcloned into pGemT-Easy (Promega, Madison, WI) and sequenced at the University of Maryland School of Medicine Biopolymer Core. The sequences of D2 products were compared to B6 sequences available in Genbank (see Additional file 2), and polymorphisms identified. When possible, unique restriction sites were identified that differentiated B6 and D2 alleles, and the corresponding restriction endonucleases were used in diagnostic digests of *Tas2r *cDNAs amplified from genomic DNA of each BXD/Ty RI strain. For *Tas2r120*, the absence of a PCR product was considered diagnostic of the D2 allele.

## Authors' Contributions

TN conducted the *in silico *analysis of the quinine taste QTL, analyzed the *Tas2r *genes, participated in the design of the study and drafted the manuscript. JB conducted the behavioral studies and QTL analysis. SM assisted with the *in silico *analysis of the quinine taste QTL and the *Tas2rs*, and with the QTL analysis. JB and SM conceived of the study, participated in its design, and edited the manuscript. All authors read and approved the final manuscript. Comments and requests should be addressed to JB or SM.

**Table 2:** Linkage of a QHCl QTL to D6Mit13 on chromosome 6.

			**1 mM qhcl**				**3 mM qhcl**			
**Chr**	**Marker**	**Position (Mb)**	**LRS**	**% Var**	***p***	**Add**	**LRS**	**% Var**	***p***	**Add**
**6**	D6Mit150	116.543	12.1	51	0.0005	0.16	11.9	50	0.00057	0.08
**6**	D6Mit254	125.974	11.4	49	0.00075	0.16	11.1	48	0.00087	0.07
**6**	D6Mit194	*126.895*	11.4	49	0.00075	0.16	11.1	48	0.00087	0.07
**6**	D6Mit61	129.173	nd	nd	nd	nd	nd	nd	nd	nd
**6**	D6Mit13	132.672	21.0	71	0.000001*	0.19	17.9	65	0.00002	0.09
**6**	D6Mit374	134.172	14.0	56	0.00018	0.17	14.3	57	0.00015	0.08
**6**	D6Mit301	136.104	14.0	56	0.00018	0.17	14.3	57	0.00015	0.08
**6**	SO6Gnf140.060	140.060	11.9	50	0.00056	0.17	12.7	53	0.00037	0.08
**8**	SO8Gnf046.785	46.785	13.4	55	0.00025	0.17	12.2	51	0.00047	0.08

Markers are listed from proximal (D6Mit150) to distal (SO8Gnf046.785), with physical position indicated in Mb. The physical position of D6Mit194 (italics) should be considered tentative. The LRS (likelihood ratio statistic) is listed for each locus, signifying the level of association of the trait (QHCl taste sensitivity) with each locus. Variance (Var) refers to the amount of the total trait variance explained by a QTL at this locus, as a percentage. Additive regression coefficients (Add) are listed for each association; in each case the coefficient is positive, indicating that D2 alleles increase the trait value (i.e. higher lick ratios). In simple marker regression analysis, all of these loci are associated with QHCl sensitivity at *p *< 0.001; only the association of sensitivity to 1 mM QHCl with D6Mit13 reaches genome-wide significance (asterisk).

## Supplementary Material

Additional File 2 Table 4: Molecular biological methods for the analysis of *Tas2rs*. Click here for file

Additional File 1 Table 3: Marker regression results for DB taste sensitivity. Click here for file

## References

[B1] Nelson G, Chandrashekar J, Hoon MA, Feng L, Zhao G, Ryba NJ, Zuker CS (2002). An amino-acid taste receptor. Nature.

[B2] Nelson G, Hoon MA, Chandrashekar J, Zhang Y, Ryba NJ, Zuker CS (2001). Mammalian sweet taste receptors. Cell.

[B3] Max M, Shanker YG, Huang L, Rong M, Liu Z, Campagne F, Weinstein H, Damak S, Margolskee RF (2001). Tas1r3, encoding a new candidate taste receptor, is allelic to the sweet responsiveness locus Sac. Nat Genet.

[B4] Montmayeur JP, Liberles SD, Matsunami H, Buck LB (2001). A candidate taste receptor gene near a sweet taste locus. Nat Neurosci.

[B5] Bachmanov AA, Li X, Reed DR, Ohmen JD, Li S, Chen Z, Tordoff MG, de Jong PJ, Wu C, West DB, Chatterjee A, Ross DA, Beauchamp GK (2001). Positional cloning of the mouse saccharin preference (Sac) locus. Chem Senses.

[B6] Li X, Staszewski L, Xu H, Durick K, Zoller M, Adler E (2002). Human receptors for sweet and umami taste. Proc Natl Acad Sci U S A.

[B7] Sainz E, Korley JN, Battey JF, Sullivan SL (2001). Identification of a novel member of the T1R family of putative taste receptors. J Neurochem.

[B8] Kitagawa M, Kusakabe Y, Miura H, Ninomiya Y, Hino A (2001). Molecular genetic identification of a candidate receptor gene for sweet taste. Biochem Biophys Res Commun.

[B9] Adler E, Hoon MA, Mueller KL, Chandrashekar J, Ryba NJ, Zuker CS (2000). A novel family of mammalian taste receptors. Cell.

[B10] Chandrashekar J, Mueller KL, Hoon MA, Adler E, Feng L, Guo W, Zuker CS, Ryba NJ (2000). T2Rs function as bitter taste receptors. Cell.

[B11] Matsunami H, Montmayeur JP, Buck LB (2000). A family of candidate taste receptors in human and mouse. Nature.

[B12] Lush IE (1984). The genetics of tasting in mice. III. Quinine. Genet Res.

[B13] Lush IE, Holland G (1988). The genetics of tasting in mice. V. Glycine and cycloheximide. Genet Res.

[B14] Lush IE, Hornigold N, King P, Stoye JP (1995). The genetics of tasting in mice. VII. Glycine revisited, and the chromosomal location of Sac and Soa. Genet Res.

[B15] Capeless CG, Whitney G, Azen EA (1992). Chromosome mapping of Soa, a gene influencing gustatory sensitivity to sucrose octaacetate in mice. Behav Genet.

[B16] Harder DB, Whitney G (1998). A common polygenic basis for quinine and PROP avoidance in mice. Chem Senses.

[B17] Bachmanov AA, Li X, Li S, Neira M, Beauchamp GK, Azen EA (2001). High-resolution genetic mapping of the sucrose octaacetate taste aversion (Soa) locus on mouse Chromosome 6. Mamm Genome.

[B18] Kim UK, Jorgenson E, Coon H, Leppert M, Risch N, Drayna D (2003). Positional cloning of the human quantitative trait locus underlying taste sensitivity to phenylthiocarbamide. Science.

[B19] Shi P, Zhang J, Yang H, Zhang YP (2003). Adaptive diversification of bitter taste receptor genes in Mammalian evolution. Mol Biol Evol.

[B20] Bufe B, Hofmann T, Krautwurst D, Raguse JD, Meyerhof W (2002). The human TAS2R16 receptor mediates bitter taste in response to beta-glucopyranosides. Nat Genet.

[B21] Behrens M, Brockhoff A, Kuhn C, Bufe B, Winnig M, Meyerhof W (2004). The human taste receptor hTAS2R14 responds to a variety of different bitter compounds. Biochem Biophys Res Commun.

[B22] Kuhn C, Bufe B, Winnig M, Hofmann T, Frank O, Behrens M, Lewtschenko T, Slack JP, Ward CD, Meyerhof W (2004). Bitter taste receptors for saccharin and acesulfame K. J Neurosci.

[B23] Pronin AN, Tang H, Connor J, Keung W (2004). Identification of ligands for two human bitter T2R receptors. Chem Senses.

[B24] Drayna D, Coon H, Kim UK, Elsner T, Cromer K, Otterud B, Baird L, Peiffer AP, Leppert M (2003). Genetic analysis of a complex trait in the Utah Genetic Reference Project: a major locus for PTC taste ability on chromosome 7q and a secondary locus on chromosome 16p. Hum Genet.

[B25] Prodi DA, Drayna D, Forabosco P, Palmas MA, Maestrale GB, Piras D, Pirastu M, Angius A (2004). Bitter taste study in a sardinian genetic isolate supports the association of phenylthiocarbamide sensitivity to the TAS2R38 bitter receptor gene. Chem Senses.

[B26] Boughter JDJ, Harder DB, Capless CG, Whitney G (1992). Polygenic determination of quinine aversion among mice. Chem Senses.

[B27] Whitney G, Harder DB (1994). Genetics of bitter perception in mice. Physiol Behav.

[B28] Blizard DA, Kotlus B, Frank ME (1999). Quantitative trait loci associated with short-term intake of sucrose, saccharin and quinine solutions in laboratory mice. Chem Senses.

[B29] Nelson TM, Munger SD, Boughter JDJ (2003). Taste sensitivities to PROP and PTC vary independently in mice. Chem Senses.

[B30] Caicedo A, Pereira E, Margolskee RF, Roper SD (2003). Role of the G-protein subunit alpha-gustducin in taste cell responses to bitter stimuli. J Neurosci.

[B31] Seto E, Hayashi Y, Mori T (1999). Patch clamp recording of the responses to three bitter stimuli in mouse taste cells. Cell Mol Biol (Noisy-le-grand).

[B32] Chen Y, Herness MS (1997). Electrophysiological actions of quinine on voltage-dependent currents in dissociated rat taste cells. Pflugers Arch.

[B33] Rosenzweig S, Yan W, Dasso M, Spielman AI (1999). Possible novel mechanism for bitter taste mediated through cGMP. J Neurophysiol.

[B34] Cummings TA, Kinnamon SC (1992). Apical K+ channels in Necturus taste cells. Modulation by intracellular factors and taste stimuli. J Gen Physiol.

[B35] Naim M, Seifert R, Nurnberg B, Grunbaum L, Schultz G (1994). Some taste substances are direct activators of G-proteins. Biochem J.

[B36] Tsunenari T, Hayashi Y, Orita M, Kurahashi T, Kaneko A, Mori T (1996). A quinine-activated cationic conductance in vertebrate taste receptor cells. J Gen Physiol.

[B37] Boughter JDJ, St John SJ, Noel DT, Ndubuizu O, Smith DV (2002). A brief-access test for bitter taste in mice. Chem Senses.

[B38] Manly KF, Cudmore RHJ, Meer JM (2001). Map Manager QTX, cross-platform software for genetic mapping. Mamm Genome.

[B39] Sheehan S (2002). BXD RI Mapping, direct data submission, MGI:2179770 (2002).

[B40] Blake JA, Richardson JE, Bult CJ, Kadin JA, Eppig JT (2003). MGD: the Mouse Genome Database. Nucleic Acids Res.

[B41] Spielman AI, Huque T, Whitney G, Brand JG (1992). The diversity of bitter taste signal transduction mechanisms. Soc Gen Physiol Ser.

[B42] Mombaerts P (2004). Genes and ligands for odorant, vomeronasal and taste receptors. Nat Rev Neurosci.

[B43] Parry CM, Erkner A, le Coutre J (2004). Divergence of T2R chemosensory receptor families in humans, bonobos, and chimpanzees. Proc Natl Acad Sci U S A.

[B44] Fischer A, Gilad Y, Man O, Paabo S (2004). Evolution of Bitter Taste Receptors in Humans and Apes. Mol Biol Evol.

[B45] Wang J, Williams RW, Manly KF (2003). WebQTL: web-based complex trait analysis. Neuroinformatics.

[B46] Knott SA, Haley CS (1992). Maximum likelihood mapping of quantitative trait loci using full-sib families. Genetics.

[B47] Boughter JD, Raghow S, Nelson TM, Munger SD (2005). Inbred mouse strains C57BL/6J and DBA/2J vary in sensitivity to a subset of bitter stimuli. BMC Genet.

[B48] Harder DB, Azen EA, Whitney G (2000). Sucrose octaacetate avoidance in nontaster mice is not enhanced by two type-A Prp transgenes from taster mice. Chem Senses.

