# Supplementary material for: Haplotypes at the Tas2r locus on distal chromosome 6 vary with quinine taste sensitivity in inbred mice
Source: BMC Genet. 2005 Jun 6;6:32. doi: 10.1186/1471-2156-6-32 (PMC1181811; doi:10.1186/1471-2156-6-32)
Supplement: Additional File 2 — Table 4: Molecular biological methods for the analysis of Tas2rs. [file 1471-2156-6-32-S2.doc]

**Table 4: Molecular biological methods for the analysis of *Tas2rs*.**

| Gene | Accession #(B6) | RestrictionEndonuclease | Forward Primer (5’ -> 3’) | **Reverse Primer (5’ -> 3’)** |  |
| --- | --- | --- | --- | --- | --- |
| | *Tas2r102* | | --- | | | AY161906 | | --- | | | *Hga* I | | --- | | | ACTCATGGGCCACTTGGTCAC | | --- | | | CAGACTCCCCCATATGACTTCC | | --- | |
| | *Tas2r103* | | --- | | | AY161917 | | --- | | | *Fok* I | | --- | | | GTTAAGAATCAGACACAGCCGTC | | --- | | | TTCCTTCCTAAATTCCTGTTCTT | | --- | |
| | *Tas2r104* | | --- | | | AY161900 | | --- | | | N/A | | --- | | | TCACACCAGATCCAGCAGAAGCC | | --- | | | ATTCCCTGCTGAACTGTGCCAG | | --- | |
| | *Tas2r105* | | --- | | | AF227147 | | --- | | | *Nhe* I | | --- | | | GAACTCAATATAGAGGACACTC | | --- | | | CCCTCATTATTATTCTAAATTACGC | | --- | |
| | *Tas2r106* | | --- | | | AY161899 | | --- | | | *Hga* I | | --- | | | TAAGCAATATTCTGCAGCAGG | | --- | | | TAAGCAATATTCTGCAGCAGG | | --- | |
| | *Tas2r107* | | --- | | | AY161898 | | --- | | | *Bsm* I | | --- | | | ATCAAGAAGCCAGTATATGGGACA | | --- | | | GAACAAGGCAGCTATTTATTTGC | | --- | |
| | *Tas2r109* | | --- | | | AY161916 | | --- | | | *Afl* III | | --- | | | GTATGTGAGAGAGTTAAGTA | | --- | | | AAGATGAAATACATGCAGTC | | --- | |
| | *Tas2r110* | | --- | | | AY161911 | | --- | | | N/A | | --- | | | GTTAGTCAGTAATGTATAAGTGGG | | --- | | | ATGTTTCCCAATGACCTCTG | | --- | |
| | *Tas2r113* | | --- | | | AY161912 | | --- | | | *Ssp* I | | --- | | | CAAATCTTCAGCAGTTACCTCAG | | --- | | | GGAAAATGATTCCTTGGTGGTCT | | --- | |
| | *Tas2r114* | | --- | | | AY161901 | | --- | | | N/A | | --- | | | GCCACTCTTTTATTGAACAGC | | --- | | | GAGCATAGTGCAAGTGAAAAGAT | | --- | |
| | *Tas2r115* | | --- | | | AY161904 | | --- | | | *Tsp* 45 I | | --- | | | TTCACCAATGGAATAGACTT | | --- | | | CAAGGTGATGCATACAATGGTC | | --- | |
| | *Tas2r116* | | --- | | | AY161910 | | --- | | | *Fnu4H* I | | --- | | | GACCTGTACATTGTTTATGGAAC | | --- | | | CTAAGATATGTAAGATCATCCAC | | --- | |
| | *Tas2r117* | | --- | | | AY161908 | | --- | | | *Tsp 45* I | | --- | | | ATGAGAATCAACTCAGAGGTTGTC | | --- | | | GTGATAATTGCAAGCAGAAATC | | --- | |
| | *Tas2r120* | | --- | | | AY161902 | | --- | | | N/A | | --- | | | GAATCATGATCTGTGAATTGAGCC | | --- | | | TAGACAGACACAATGAATTTGG | | --- | | | | TCAGTCAGCATCTCATCTGCCTC | | --- | | GACCTGTTTAAGTTTCTGGTTTCCC | |
| | *Tas2r121* | | --- | | | AY161903 | | --- | | | *Sfc* I | | --- | | | GGTCAGTCACTACATAAGAAGG | | --- | | | GTCATCACCCAAAGACTGGC | | --- | |
| | *Tas2r122* | | --- | | | AX097842 | | --- | | | *Msp* I | | --- | | | GGATTCACACATCTACATCAATGTGGC | | --- | | | GAAATCCATCAGTTATATTGAGTGTA | | --- | |
| | *Tas2r123* | | --- | | | AY161909 | | --- | | | *Aci* I | | --- | | | TATGTAGACTGCTTAAATGCATC | | --- | | | CTATGAACTGATCATCCTCAAGA | | --- | |
| | *Tas2r124* | | --- | | | AY161905 | | --- | | | *Afl* II | | --- | | | GCAGTCATATAGAATTTGGGCT | | --- | | | CCCTGCTACTCCATGTCAAG | | --- | |
| | *Tas2r125* | | --- | | | AY161913 | | --- | | | *Tsp 45* I | | --- | | | CAGCGGACTTACACAAATCAAGC | | --- | | | CTTCACTACTAAAGAGCATTGACAGAG | | --- | |
| | *Tas2r129* | | --- | | | AY161914 | | --- | | | *Fok* I | | --- | | | CCTTACAGACAATGGATGGAATCG | | --- | | | TAGAATATGCAAGATGATCTGC | | --- | |
| | *Tas2r130* | | --- | | | AY161897 | | --- | | | *Tsp 45* I | | --- | | | GATCGTGTCTTCCTTTCTCT | | --- | | | GAACCCTAAACAGCCAAGTG | | --- | |
| | *Tas2r131* | | --- | | | AY161915 | | --- | | | *Afl* II | | --- | | | GGATGATTTTCTGTGGAAGTTTG | | --- | | | CAGTGACCTTCCAAGTCTCCCTG | | --- | |
| | *Tas2r136* | | --- | | | AY161907 | | --- | | | *Xba* I | | --- | | | GAATACAATGCTACAGACCCTGC | | --- | | | AATGAAGACCACATGAATCAGTG | | --- | |
| | *Tas2r140* | | --- | | | AY161918 | | --- | | | *Sfc* I | | --- | | | GTCCCCTTGTAGACTATGGAA | | --- | | | GAAGATTACATTAAGGACTTGG | | --- | |

Individual mouse *Tas2rs* genes are named based on the Mouse Genome Informatics (MGI) nomenclature, and are listed in numerical order. Accession numbers are for the B6 alleles. Restriction endonucleases used for diagnostic digestion of *Tas2r* PCR products are listed, where applicable (no unique restriction sites were identified for *Tas2r104*, *Tas2r110*, *Tas2r114* or *Tas2r120*). Forward and reverse PCR primers for each *Tas2r* are indicated (two different pairs were used for *Tas2r120*), and are based on the B6 alleles indicated in the second column.
