# Supplementary material for: Haplotypes at the Tas2r locus on distal chromosome 6 vary with quinine taste sensitivity in inbred mice
Source: BMC Genet. 2005 Jun 6;6:32. doi: 10.1186/1471-2156-6-32 (PMC1181811; doi:10.1186/1471-2156-6-32)
Supplement: Additional File 1 — Table 3: Marker regression results for DB taste sensitivity. [file 1471-2156-6-32-S1.doc]

**Table 3: Marker regression results for DB taste sensitivity.**

|  | | | | **1 mM DB** | | | | **3 mM DB** | | | |  |
| --- | --- | --- | --- | --- | --- | --- | --- | --- | --- | --- | --- | --- |
| **Chr** | **Marker** | **Position**  **(Mb)** | **LRS** | | **% Var** | ***p*** | **Add** | **LRS** | **% Var** | ***p*** | **Add** | |
| **2** | D2Mit223 | 134.736 | 11.2 | | 48 | 0.0008 | -0.16 | 3.9 | 21 | 0.048 | -0.06 | |
| **2** | D2Msw142 | 140.718 | 11.6 | | 49 | 0.0007 | -0.16 | 7.3 | 36 | 0.0068 | -0.08 | |
| **8** | S08Gnf124.650 | 122.875 | 13.0 | | 53 | 0.0003 | -0.20 | 7.9 | 37 | 0.005 | -0.09 | |
| **12** | D12Mit88 | 49.862 | 11.9 | | 50 | 0.0006 | -0.17 | 0.8 | 5 | 0.3734 | 0.05 | |
| **12** | D12Mit285 | 49.872 | 11.9 | | 50 | 0.0006 | -0.17 | 0.8 | 5 | 0.3724 | 0.05 | |
| **12** | D12Mit36 | 55.861 | 10.4 | | 46 | 0.0013 | -0.16 | 2.5 | 14 | 0.112 | -0.05 | |
| **12** | S12Gnf062.520 | 59.639 | 10.4 | | 46 | 0.0013 | -0.16 | 2.5 | 14 | 0.112 | -0.05 | |

Associations with markers on chromosomes 2, 8, and 12 are shown; physical position is indicated in Mb. The LRS is listed for each locus, signifying the level of association of the trait (DB taste sensitivity) with each locus. Variance (Var) refers to the amount of the total trait variance explained by a QTL at this locus, as a percentage. Additive regression coefficients (Add) are listed for each association; in most cases the coefficients are negative, indicating that B6 alleles increase the trait value (i.e. higher lick ratios). In simple marker regression analysis, these loci provided the greatest level of association with sensitivity to 1 mM DB (*p* < 0.003) and met the genome-wide criteria for “suggestive” (LRS > 9.4) but not significance (LRS > 17.2). None of these associations reached criteria for suggestive (LRS > 9.4) or significance (LRS > 15.6) at 3 mM DB.
